# Extensively Drug-Resistant Tuberculosis: A Sign of the Times and an Impetus for Antimicrobial Discovery

**DOI:** 10.3390/ph3072268

**Published:** 2010-07-20

**Authors:** Shelley E. Haydel

**Affiliations:** Biodesign Institute Center for Infectious Diseases and Vaccinology, School of Life Sciences, Arizona State University, Tempe, AZ, 85287-5401, USA; E-Mail: Shelley.Haydel@asu.edu; Tel.: +1-480-727-7234; Fax: +1-480-727-0599

**Keywords:** tuberculosis, antimicrobial, drug, resistance

## Abstract

*Mycobacterium tuberculosis* is an extraordinarily successful human pathogen, infecting one-third of the world’s population and causing nearly two million deaths each year. In this article, current trends in worldwide tuberculosis (TB) incidence, prevalence, and mortality are discussed along with standard TB treatment regimens, characteristics of first-line and second-line anti-tuberculosis drugs, and mechanisms of antibiotic resistance. The global TB emergency has been further exacerbated by extensively drug-resistant (XDR) TB strains that are resistant to our best antibiotics and very difficult to treat. This review also focuses on the emergence of XDR-TB strains, the global health impact, and existing treatment options and outcomes for XDR-TB disease. Finally, this review briefly describes new anti-tuberculosis drugs currently in Phase II clinical evaluations and the impetus for discovering new antibacterial compounds to target drug-resistant *M. tuberculosis* and improve tuberculosis therapy.

## 1. Introduction

Tuberculosis (TB) is believed to have been present in humans for thousands of years, as evidenced by the bones of ancient Egyptian mummies showing deformities consistent with the disease. Historically, pulmonary TB was known as the “Great White Plague” (causing about one in four deaths) of the 17th and 18th centuries in Europe, “phthisis” (a Greek term meaning to waste away), “scrofula” (swollen glands of the neck), and “consumption” (progressive wasting of the body) [1]. From 1700 to 1900, it is estimated that TB was responsible for one billion deaths and killed more people than any other disease [1,2]. Prior to the introduction of antibiotics in the 1950s, improved sanitation and living conditions significantly reduced the incidence of TB disease.

TB is transmitted via the respiratory route as a highly infectious aerosol with varying outcomes occurring from this initial *Mycobacterium tuberculosis* exposure. These outcomes can range from immediate organism destruction by the host’s immune system to infected individuals developing active primary TB disease within 1–3 years [3]. However, the majority of individuals infected with *M. tuberculosis* have a non-contagious, clinically-latent infection with an absence of clinical symptoms [4]. Latently-infected individuals have a 5–10% risk of developing reactivation TB disease during their lifetime [3], often due to immunosuppressive circumstances, with HIV infection being the greatest identified cause [5].

The discovery of the first antibiotic, streptomycin, to treat TB in 1944 was soon met with the appearance of the first antibiotic-resistant *M. tuberculosis* isolates [6]. A rapid succession of additional anti-TB agents was discovered in the late 1940s through the 1950s, with the last, rifampin, discovered in the 1960s [7]. To circumvent the development and emergence of drug resistance, a multidrug chemotherapeutic approach for treating TB was also introduced in the 1960s [8,9,10]. The concomitant emergence of HIV in 1980s further complicated and worsened the ongoing TB epidemic [11], and the global TB/HIV coinfection epidemic continues today with TB infecting one-third of the 33.2 million people living with HIV [12]. Continued evolution and exacerbation of the drug resistance problem has led to extensively drug-resistant TB, or XDR-TB, which is resistant to first-line and second-line TB antibiotics [13,14]. MDR and XDR forms of TB are extraordinarily difficult to treat, particularly in immunocompromised patients, and pose serious threats to global health. 

## 2. Global Incidence, Prevalence, and Mortality of TB

Worldwide, TB is the second leading cause of death from a single infectious agent. Highlighting its success as a human pathogen, the World Health Organization (WHO) declared TB a global emergency in 1993 and warned that the disease could kill 30 million people over the next decade if effective control efforts were not implemented. While significant epidemiological, treatment, and control strategies have been employed over the past decade, the WHO estimate of nearly 30 million deaths was relatively accurate, with TB continuing to be one of the world’s major infectious diseases. Globally, one-third of the world’s population is infected with TB, with over nine million incident cases of active TB disease and an estimated 1.3 million deaths occurring per year [15]. Regionally, the majority of TB cases in 2008 existed in Asia (55%) and Africa (30%), with significantly lower proportions of cases in the Eastern Mediterranean (7%), Europe (5%), and the Americas (3%) [16]. Given the vast number of individuals who succumb to the disease each year, long-term actions to fight this public health challenge must be and are aimed at reducing global mortality and eliminating the disease [17,18].

Global control efforts over the past decade have resulted in relative stabilization of incident and prevalent cases of TB and reduction in TB deaths (Table 1) [19]. In 2004, global incidence rates peaked at 142 cases per 100,000 population, and the slow rate of decline since 2004 has been at less than 1% per year [19]. Accounting for population growth, the currently estimated 139 incident cases per 100,000 population has remained relatively stable for the past several years [16,19,20]. Prevalence is a direct indicator of the global TB burden, referring to the number of individuals with the disease at a particular time [21,22]. The overall numbers of estimated prevalent cases of TB decreased from 13.9 to 11.1 million from 2006 to 2008, corresponding to a reduction in the number of cases per capita from 210 to 164 per 100,000 population in 2006 and 2007, respectively (Table 1) [16,19]. Globally, TB treatment success rates in 2007 were 86%, thus meeting the 85% success rate target first established by the WHO in 1991 [15,16,18]. 

Worldwide, TB accounts for approximately one-fourth of HIV-related deaths and is the leading cause of death in HIV-infected adults in developing countries [12,16,22,23]. In 2000, nine percent of all new TB cases and 12 percent of TB cases in adults were attributable to HIV infection [22]. In 2008, the influence of the HIV pandemic on the global burden of TB continued to escalate as an estimated 1.4 million incident cases, representing 15% of the total TB incidence, occurred in HIV-positive patients [16]. While the overall number of HIV-related or HIV-attributed TB deaths has remained relatively stable over the past decade (Table 1), the interlinked TB and HIV epidemics continue to inflect serious morbidity and mortality in sub-Saharan African countries [24]. Approximately 78% of the globally estimated 1.4 million HIV-positive TB patients live in sub-Saharan Africa [12] with 30 to 40% of these HIV-infected individuals dying from TB [25].

**Table 1:** Estimated global incidence, prevalence, and mortality of TB [16,19,22,26].

	2000	2006	2007	2008
** Incidence^a^**	8.3 million	9.24 million	9.27 million	9.4 million
** Prevalence^b^**	16.6 million^c^	13.9 million	13.7 million	11.1 million
**Deaths (total)**	1.8 million	1.7 million	1.7 million	1.8 million
**HIV-negative**	1.6 million	1.5 million	1.3 million	1.3 million
**HIV-positive**	0.226 million	0.231 million	0.456 million^d^	0.52 million^d^

^a^ Incident cases refer to the estimated number of new cases reported within a given year. ^b^ Prevalent cases refer to the estimated number of cases which exist within a population within a given year [21,22]. ^c^ In 2000, the global prevalence was estimated as twice the incidence [22].^d^ Increased numbers of HIV-positive cases result from new data from provider-initiated HIV testing, not doubling between 2006 and 2007 [16,19].

## 3. Recommended TB Treatment and the Current Arsenal of TB Antibiotics

The current standard chemotherapeutic regimen for treating new pulmonary TB patients consists of a multidrug combination of the first-line anti-TB drugs-isoniazid, rifampin, pyrazinamide, and ethambutol-administered for an intensive, initial period of two months. A continuation phase of treatment for an additional four months consists of administration of isoniazid and rifampin. In countries reporting high levels of isoniazid resistance occurring in new TB patients or lacking isoniazid drug susceptibility results, ethambutol is administered throughout the four-month continuation phase of treatment [27]. Upon availability of drug susceptibility profiles and confirmed sensitivity of isoniazid, administration of ethambutol may be omitted in the continuation phase [27,28]. 

**Figure 1:**
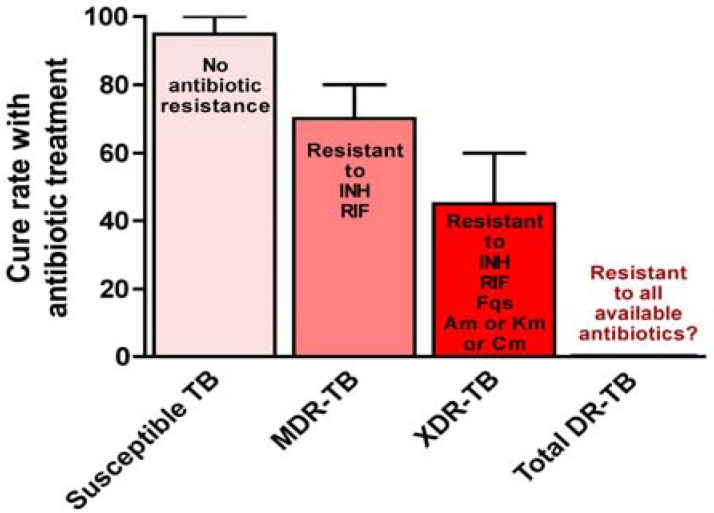
Antibiotic cure rates of TB based on drug resistance patterns. Estimated cure rates for antibiotic-susceptible TB, MDR-TB, and XDR-TB in HIV-negative patients are >95%, 60–80%, and 30–60%, respectively [60,61,62,63]. Although TB strains exhibiting resistance to all first-line and second-line antibiotics have not been identified, total drug-resistant (DR)-TB would be untreatable with existing chemotherapeutic agents. INH, isoniazid; RIF, rifampin; Fqs, fluoroquinolones; Am, amikacin; Km, kanamycin; Cm, capreomycin.

The implementation of the Directly Observed Treatment, Short course (DOTS) strategy by the WHO in the 1990s elicited great effectiveness in TB control, achieved cure rates of nearly 80% [29], and was subsequently expanded as an internationally recommended approach for TB control [30,31]. DOTS consists of a broad TB control effort focused upon five principal elements: (*i*) political commitment for financing; (*ii*) proper case detection with appropriate microbiological laboratory support; (*iii*) standard chemotherapeutic treatment with patient support and supervision, which includes directly observed therapy; (*iv*) consistent availability of effective drugs; and (*v*) standard monitoring and evaluation system with impact measurements. Supervised administration of antibiotics during the intensive phase of therapy, in accordance with the recommended DOTS strategy, results in a greater than 85% cure rate of new, antibiotic-susceptible TB cases (Figure 1) [30,32]. The DOTS supervised program facilitates patient adherence to the treatment regimen and maximizes the likelihood of treatment completion [28].

First-line anti-TB antibiotics target actively replicating *M. tuberculosis* cells in the lung (Table 2) and significantly reduce transmission rates of *M. tuberculosis* to other persons within the first two months of treatment. The bactericidal antibiotics, isoniazid and rifampin, are active against dividing cells with rifampin also having activity against dormant bacteria, thus accounting for sterilizing properties during the short-course antibiotic regimen (Table 2) [33,34,35]. Pyrazinamide exhibits greatest activity against dormant organisms localized within macrophages or the acidic environment of the pulmonary caseous lesion [36]. Inclusion of ethambutol in the first-line drug regimen is recommended to prevent rifampin resistance when isoniazid resistance is suspected [28].

Second-line antibiotics are introduced into treatment regimens when resistance to primary antibiotics emerges. However, secondary antibiotics exhibit lower potency and/or greater toxicity [38]. The fluoroquinolone, aminoglycoside, and capreomycin antibiotics target DNA replication and protein synthesis and offer the greatest effectiveness of the second-line anti-TB drugs (Table 3) [39,40]. The remaining antibiotics exhibit bacteriostatic activity (Table 3) and are considerably less potent, more toxic, and more expensive [38]. Fortunately, in 2000, the WHO and its partners established the Green Light Committee Initiative which allows countries access to concessionally-priced, second-line anti-TB drugs for treating individuals with MDR-TB in accordance with WHO guidelines [18,41,42]. 

Therapy for treating TB has not always been mediated via chemotherapeutic agents. Prior to the availability of effective antibiotics, surgical intervention was an important form of therapy for pulmonary TB [43]. In this day and age with the emergence of MDR- and XDR-TB, surgical pulmonary resection is once again considered effective management for the treatment of patients with drug-resistant TB [43,44,45,46,47,48]. Studies have also shown that administration of antibiotics along with surgical management of pulmonary MDR-TB result in improved outcomes compared to surgical intervention alone [43].

**Table 2:** First-line anti-TB drugs [37].

First-line antibiotics	Antibiotic class/structure	Delivery route	Activity	Mechanism of action	Genes and gene products associated with resistance
Isoniazid	Pyridine hydrazide	Oral	Bactericidal	Inhibits mycolic acid (cell wall) synthesis	*katG*; catalase-peroxidase
					*inhA*; enoyl-ACP reductase
					*ndh*; NADH dehydrogenase II
Rifampin	Rifamycin	Oral	Bactericidal	Inhibits RNA synthesis	*rpoB*; β-subunit of RNA polymerase
Pyrazinamide	Nicotinamide analog	Oral	Bacteriostatic/ bactericidal	Disrupts cell membrane energetics and inhibits membrane transport	*pncA*; nicotinamidase/pyrazinamidase
Ethambutol	Ethylenediamine derivative	Oral	Bacteriostatic	Inhibits arabinogalactan (cell wall) synthesis	*embCAB*; arabinosyl transferase

## 4. Emergence and Global Health Impact of XDR-TB

The TB global emergency is further complicated by MDR- and XDR-TB strains that are resistant to our best antibiotics, very difficult to treat, and associated with greater morbidity and mortality than antibiotic-susceptible TB (Figure 1). An individual may develop the drug resistant form of TB via inadequate therapy that enables the selection of drug-resistance (acquired resistance) or infection with a drug-resistant TB strain (primary resistance) [49]. While infection with an exogenous drug-resistant TB strain is related to infection control measures, the development of acquired *M. tuberculosis* resistance is multi-faceted and can be attributed to various social, political, economic, epidemiological, and pathophysiological factors [50]. Certainly, scientists investigate the cellular and molecular mechanisms to explain the development of drug-resistant TB strains, but other influences including, but not limited to, improper or poor health management practices or infrastructure, inadequate therapeutic regimens, antibiotic misuse, insufficient or unobtainable resources, poor socioeconomic conditions, individual immunocompetence, patient compliance, and complicated personal issues have also played roles in the evolution and progression of antibiotic resistance [38,50,51,52,53,54,55].

**Table 3:** Second-line anti-TB drugs [37].

Second-line antibiotics	Antibiotic class/structure	Delivery route^a^	Activity	Mechanism of action	Genes and gene products associated with resistance
Streptomycin	Aminoglycoside	IM injection	Bactericidal	Inhibits protein synthesis	*rpsL*; S12 ribosomal protein **
					*rrs* ; 16S rRNA
Kanamycin/Amikacin	Aminoglycoside	IM injection	Bactericidal	Inhibits protein synthesis	*rrs*; 16S rRNA
Capreomycin	Polypeptide	IM injection	Bactericidal	Inhibits protein synthesis	*rrs*; 16S rRNA **
					*tlyA* ; putative rRNA methyltransferase
Levofloxacin	Fluoroquinolone	Oral or IV	Bactericidal	Inhibits DNA replication	*gyrA*; DNA gyrase subunit A
Moxifloxacin	Fluoroquinolone	Oral or IV	Bactericidal	Inhibits DNA replication	*gyrA*; DNA gyrase subunit A
Gatifloxacin	Fluoroquinolone	Oral or IV	Bactericidal	Inhibits DNA replication	*gyrA*; DNA gyrase subunit A
Ethionamide	Thioamide	Oral	Bacteriostatic	Inhibits mycolic acid (cell wall) synthesis	*inhA*; enoyl-ACP reductase **
					*etaA/ethA* ; flavin monooxygenase
Cycloserine	Isoxazolidinone	Oral	Bacteriostatic	Inhibits peptidoglycan (cell wall) synthesis	unknown (*alrA*; D-alanine racemase in *Mycobacterium smegmatis*)
Para-aminosalicylic acid	Salicyclic acid	Oral	Bacteriostatic	Inhibits folic acid synthesis	*thyA*; thymidylate synthase

^a^ IM, intramuscular; IV, intravenous.

MDR-TB strains exhibit antibiotic resistance to two of the best first-line anti-TB antibiotics, isoniazid and rifampin, while XDR-TB is defined as being resistant to isoniazid, rifampin, to any fluoroquinolone, and one or more of three injectable anti-TB antibiotics (capreomycin, kanamycin, and amikacin) [56]. Removal of the fluoroquinolones and the injectable drugs from the TB treatment arsenal means losing the most potent and least toxic second-line antibiotic treatment options. Considering that XDR-TB is resistant to powerful first-line and second-line antibiotics, patients must be treated with more expensive, less effective second-line antibiotics, resulting in a longer treatment course for a minimum of 18–24 months, lower cure rates (Figure 1), and significantly increased healthcare costs [57]. Moreover, second-line therapeutic treatment requires strict patient monitoring, supervision, counseling, and support to prevent further drug resistance that could potentially render the disease untreatable (Figure 1) [14,58].

In 2004, there were an estimated 424,000 MDR-TB cases, representing a 64% increase from the global number of nearly 273,000 MDR-TB cases estimated in 2000 [59]. For the past several years, the number of worldwide MDR-TB cases consistently remained around 450,000 per year. XDR-TB, first described in March 2006 [13], generally evolves from MDR strains. The WHO estimates 30,000 cases of XDR-TB per year, but the number of countries or territories reporting at least one case of XDR-TB has nearly tripled from 20 in 2007 to 57 in 2009, thus emphasizing its spread [16,19]. On April 1, 2009, WHO officials stated that XDR-TB is “a very deadly and devastating epidemic” that is “poised to grow much worse very quickly.” The warning signs are certainly present, and without action, we are facing a disease epidemic that will undoubtedly continue without new drugs, diagnostics, and vaccines to combat it.

## 5. Genetic Basis of *M. tuberculosis* Antibiotic Resistance

Efforts to understand the molecular basis of *M. tuberculosis* antibiotic resistance have advanced significantly and investigations of potentially unique genetic traits in MDR- and XDR-TB strains are ongoing. Unlike other bacterial pathogens, there is no evidence that gene acquisition contributes to antibiotic resistance in *M. tuberculosis* [64]. The mutated genes and gene products associated with *M. tuberculosis* drug resistance are included in Table 2 and Table 3. Isoniazid resistance is linked to alterations in the catalase-peroxidase gene (*katG*), the *inhA* gene, which encodes an enzyme involved in mycolic acid biosynthesis, or the NADH dehydrogenase II gene (*ndh*) [65,66,67,68]. Genetic mutations in *rpoB*, which encodes the RNA polymerase β-subunit, result in rifampin resistance [69,70,71]. Mutations in *pncA* that eliminate pyrazinamidase/nicotinamidase activity confer pyrazinamide resistance [72,73]. Numerous genetic mutations present within the *embCAB* operon, which facilitates production of arabinosyl transferase, have been linked to ethambutol resistance [74,75], but other genes may also be involved [76].

Similar to the first-line TB antibiotics, genetic mutations account for all known mechanisms of resistance for the second-line TB drugs (Table 3). Streptomycin resistance is associated with mutations in the *rpsL*, ribosomal S12 protein, and *rrs*, 16S rRNA, genes [77,78,79,80]. Similar to streptomycin, kanamycin and amikacin resistance are linked to genetic mutations that occur within the *rrs* gene with evidence of cross-resistance occurring between kanamycin and capreomycin or viomycin in mycobacteria [81,82,83,84,85,86]. Mutagenesis of the *tlyA* gene, which has homology to rRNA methyltransferases, also confers capreomycin resistance [87]. Clinical resistance to the quinolone family antibiotics, levofloxacin, moxifloxacin, and gatifloxacin, is attributed to mutations occurring within the *gyrA* gene encoding DNA gyrase [88,89]. Ethionamide resistance is linked to *inhA* mutations, whereby cross-resistance occurs between isoniazid and ethionamide [66,90], and to mutations in the *etaA* (*ethA*) gene, which codes for flavin monooxygenase responsible for activation of ethionamide [91,92]. Recent evidence links para-aminosalicylic acid resistance to mutations within the *thyA* gene, which produces thymidylate synthase A, but mechanisms or targets independent of thymine nucleotide biosynthesis are also likely involved [93,94]. While inactivation of the *alrA* gene, encoding D-alanine racemase, causes increased sensitivity to cycloserine in *Mycobacterium smegmatis* [95] and overexpression of *alrA* confers mycobacterial resistance to cycloserine [96], the genetic mechanism of cycloserine resistance in *M. tuberculosis* is currently unknown.

While accumulation of genetic mutations associated with first-line and second-line drug resistance is implicated in TB drug resistance, efforts to delineate molecular insight into MDR- and XDR-TB-specific genetic traits are underway. Comparative computational analysis of drug-sensitive *M. tuberculosis* and MDR- and XDR-TB KwaZulu-Natal strains (discussed below) suggested that drug resistance may be linked to single-nucleotide variations (348 common to all KwaZulu-Natal strains) which could influence drug susceptibility patterns and protein function [97]. Whole-genome sequencing of MDR- and XDR-TB outbreak strains in HIV-infected patients from KwaZulu-Natal revealed novel mutations not previously associated with drug resistance [98]. However, the study further determined that the majority of mutations came from a common ancestor, suggesting that XDR-TB strains can evolve without fitness changes or XDR-specific mutations [98].

## 6. Existing Treatment Regimens and Treatment Outcomes for XDR-TB

While MDR-TB can be effectively treated with a long-term regimen of second-line antibiotics [99], XDR-TB is often considered very difficult to treat, or is even untreatable, with existing chemotherapeutic agents [100,101]. A large retrospective study revealed that XDR-TB cases have a worse clinical outcome than MDR-TB cases resistant to all first-line antibiotics (39% *vs*. 54% treatment success, respectively) [102]. Importantly, susceptibility to at least one first-line drug increased treatment success of MDR-TB cases (67%), indicating that the XDR-TB designation can be a strong predictor of poor treatment outcomes [102]. Sytematic review and meta-analysis of treatment outcomes of over 8,000 patients with MDR-TB (data from 33 studies in 20 countries) determined that treatment success improved substantially when patients were treated for at least 18 months with directly observed therapy occurring throughout the entire treatment period [103,104].

XDR-TB cases with resistance patterns corresponding to the WHO definition (resistance to isoniazid, rifampin, to any fluoroquinolone, and one or more of three injectable anti-TB antibiotics) are usually not identified during clinical treatment since second-line drugs are administered upon the likelihood of first-line antibiotic resistance [102]. Moreover, because of the recent classification of XDR-TB within the past five years, patients with XDR-TB are often not differentiated from individuals with MDR-TB prior to the initiation of treatment. Therefore, most existing studies focus on retrospective identification of XDR-TB isolates from patients diagnosed with MDR-TB. Although treatment successes vary among existing studies, poor treatment outcomes appear to be invariably associated with XDR-TB (Figure 1, Table 4) [102].

A startling 2006 study revealed an XDR-TB outbreak in 53 HIV-positive patients which resulted in 52 deaths occurring within 16 days of TB diagnosis (Table 4) [105]. These patients from the province of KwaZulu Natal in the Republic of South Africa, which has a high incidence of TB (940 cases per 100,000 population) and high prevalence of HIV infection (19% of the adult population), likely had severe immunosuppression, had not previously received treatment for TB, and were not known to have acquired or been treated for resistant TB disease [49,105]. From June 2005 through March 2007 in the town of Tugela Ferry, located in central KwaZulu-Natal Province, a total of 217 XDR-TB cases were identified, yielding a striking mortality rate of 84% [106]. Retrospective assessments indicated that hospital and health system failures, inefficient airborne infection control in health care facilities, and inadequate knowledge and understanding of clinicians in KwaZulu-Natal province of South Africa influenced the high mortality and high transmission rates of this XDR-TB outbreak [49,107]. Moreover, in these HIV-positive patients, exogenous reinfection with MDR- or XDR-TB strains, rather than acquired drug resistance, was implicated in the development of this outbreak [49]. Most importantly, these reports and others in non-HIV-infected patients revealed the rapid lethality of XDR-TB and alerted public health officials of the emerging epidemic and global health significance of XDR-TB strains [57,102,108,109,110,111].

While HIV coinfection will result in poor treatment outcomes for XDR-TB patients, treatment success rates in HIV-negative, XDR-TB patients are quite variable, ranging from 29–60% (Table 4) [62,112]. In a retrospective study of South Korean XDR-TB patients treated with a median number of four anti-TB drugs (range, 0–16), the treatment success rate was 29% [63]. Patients were treated with individualized regimens based on antibiotic susceptibility profiles and direct observed therapy was only implemented upon admission into a national TB hospital [63].

**Table 4:** XDR-TB treatment outcomes.

Study	Study location	XDR-TB patients	HIV status	Treatment success rate
Gandhi *et al*. [105]	KwaZulu Natal, Republic of South Africa	53	positive	2%
Migliori *et al*. [102]	Europe (Estonia, Germany, Italy, and Russian Federation)	64	negative	39%
Keshavjee *et al*. [62]	Tomsk, Russia	29	negative	48%
Chan *et al*. [57]	United States	10	negative	50%
Mitnick *et al*. [112]	Peru	48	negative	60%
Kim *et al*. [63]	South Korea	75	negative	29%

As reported by Keshavjee *et al*. [62] and Mitnick *et al*. [112], more aggressive chemotherapeutic treatment approaches which incorporate the use of at least five antibiotics with demonstrated isolate susceptibility have resulted in improved treatment success rates. Aggressive treatment of HIV-negative patients with XDR-TB in Tomsk, Russia resulted in successful treatment of 14/29 (48%) patients [62]. Most XDR-TB strains in the Tomsk, Russia cohort study were resistant to all first-line antibiotics and ethionamide [62]. Although all drugs were administered under direct observation and adherence rates were similar, treatment failure was more commonly associated with XDR-TB than with non-XDR-TB (31% *vs*. 8.5%, respectively) [62]. Using a similar, aggressive treatment approach with daily, supervised therapy of at least five antibiotics including cycloserine, an injectable drug, and a fluoroquinolone administered at the highest tolerated doses, 29/48 (60%) of HIV-negative, XDR-TB patients in Peru were successfully treated [112]. These curative rates were comparable to the 400/603 (66%) MDR-TB patients who successfully completed treatment [112]. These encouraging results demonstrate that XDR-TB patients can be cured at respectable rates and that the disease does not automatically result in a death sentence. Incorporation of a comprehensive treatment approach with an effective and well-managed TB control program, implementation of aggressive drug treatment strategies, and further development of appropriate chemotherapeutic interventions are all deemed critical for improving treatment outcomes, eliminating the spread of XDR-TB, and avoiding the prospect of total drug-resistant TB strains [112].

## 7. The Inherent Need for New Anti-TB Drugs

For the most part, TB therapy has remained unchanged for nearly four decades and often consists of taking more than 10 pills per day for a minimum of six months for antibiotic-susceptible disease [113]. The diagnosis of MDR-TB or XDR-TB further subjects the patients to as many as 20 pills per day, as well as antibiotic intramuscular injections, for 18 to 24 months. This lengthy treatment is not only more expensive than first-line antibiotics, but also comes with devastating, toxic side effects, emotional and social anxieties, and pyschological stresses [114,115,116,117]. With XDR-TB treatment success rates ranging from 30–60% in HIV-negative patients (Figure 1, Table 4), XDR-TB strains threaten to return TB treatment to the pre-antibiotic era, when more than 50% of TB patients succumbed to the disease [113].

The spread of XDR-TB and the poor treatment outcomes in both developing and developed countries clearly indicate that XDR-TB knows no boundaries. Therefore, its emergence and spread potentially jeopardizes our abilities to fight the disease in all people, irrespective of geographic location, thus posing an incredible global threat to public health. While preserving the effectiveness of the existing first-line and second-line antibiotics is ideal, the mere existence of XDR-TB strains suggests that we are embarking upon an ominous era whereby totally drug-resistant TB strains could evolve. In order to combat MDR- and XDR-TB and the overall spread of antibiotic resistant TB strains, the need for new anti-TB drugs is imminent. 

## 8. Development of Novel Chemotherapeutic Anti-TB Compounds

After discovery and development of new anti-TB drugs flourished in the mid-1900s, the TB drug pipeline was reduced to a mere leaky faucet with the discovery of new classes of antibiotics being virtually nonexistent. It has been more than 40 years since the last novel TB-specific antibiotic was introduced into clinical practice. Given the challenge of treating XDR-TB, fortunately, the existing pipeline for new classes of anti-TB drugs shows promise. While there are a number of propitious candidates currently in various stages of discovery and clinical development [118,119,120], the new anti-TB compounds described herein represent drugs with novel structures and/or mechanisms of action currently in Phase II clinical evaluations (Table 5). 

A novel diarylquinoline, TMC207 (also known as R207910), exhibits potent *in vitro* bactericidal activity against aerobically-replicating, drug-sensitive and MDR *M. tuberculosis* [121] as well as dormant, antibiotic-susceptible *M. tuberculosis* [122]. Importantly, TMC207 also demonstrates rapid mycobactericidal activity in experimentally-infected animals [121] and in patients with drug-susceptible or MDR pulmonary TB [123,124]. The diarylquinoline, TMC207, offers a new mechanism of anti-TB action by specifically inhibiting the mycobacterial ATP synthase, thus diminishing bacterial energy production in the form of ATP molecules [121].

**Table 5:** Novel anti-TB drugs currently in Phase II clinical trials.

Drug	Class	Mechanism of action
TMC207	Diarylquinoline	Inhibits ATP synthase and energy metabolism
PA-824	Nitroimidazo-oxazine	Inhibits mycolic acid synthesis
OPC-67683	Nitrodihydro-imidazooxazole	Inhibits mycolic acid synthesis

Another class of promising compounds with anti-TB activity is the nitroimidazoles, including PA-824 and OPC-67683. Although the exact mechanism of their action is not completely understood, the PA-824 prodrug requires activation by bacterial dehydrogenase and nitroreductase to inhibit mycolic acid synthesis [125,126,127]. Mycolic acids are important constituents of the mycobacterial cell wall, are involved in pathogenicity, and exhibit diverse immunological functions [128]. PA-824 displays strong bactericidal activity against replicating and non-replicating *M. tuberculosis* and exhibits bactericidal activity when administered orally to experimentally-infected animals [125,129,130]. Of particular significance, a regimen of PA-824, moxifloxacin, and pyrazinamide demonstrated bactericidal and sterilizing activity against TB in experimentally-infected mice, suggesting efficacy against MDR-TB [131]. Additional safety, tolerability, and pharmacokinetic studies with PA-824 in healthy human subjects are ongoing [132,133]. The OPC-67683 dihydroimidazo-oxazole also demonstrates very potent *in vitro* and *in vivo* bactericidal activity against antibiotic-sensitive and MDR *M. tuberculosis* [134,135,136]. While the specific bacterial target of OPC-67683 is not yet known, the compound inhibits production of cell wall mycolic acids [134], suggesting a similar mechanism of action as PA-824. Unlike metronidazole (another nitroimidazole compound), which kills dormant *M. tuberculosis* under anaerobic conditions [137], OPC-67683 and PA-824 are bactericidal against *M. tuberculosis* grown in either aerobic or anaerobic states. The ability to target both actively replicating and dormant bacteria allows OPC-67683 and PA-824 to function as double-edged swords and could potentially shorten the duration of TB treatment [126].

## 9. Future Perspectives Regarding the Discovery of New Anti-TB Compounds

Fortunately, there has been a resurgence in TB drug research over the past decade, and numerous compounds are progressing through the various stages of the pharmaceutical discovery and development pipeline. Efforts by the Stop TB Partnership and the TB Alliance have been instrumental in stimulating and advancing TB research and accelerating the discovery and development of new drug therapies for treating TB. For a comprehensive list of compounds and the current phase of discovery or development, see the Stop TB Partnership Working Group on New TB Drugs (http://www.stoptb.org/wg/new_drug) and the TB Alliance (http://www.tballiance.org). Although several new anti-TB drugs have advanced to preclinical development and/or clinical trials, the timeline for their potential introduction into clinical practice and the impact on TB treatment are difficult to predict. 

Drug discovery and development is a very complex and expensive process with the estimated costs between $800 million and $1 billion to bring a new drug to market [138,139]. Despite this daunting process, a continuation of the basic research and discovery processes is critical to identify new families of anti-TB agents targeting novel enzymes and/or cell processes associated with viability and/or bacterial virulence [118,120]. Exploration of novel targets and mechanisms of action are necessary to discover candidate compounds with efficacy against drug-sensitive and drug-resistant TB, with activity against active and latent TB, and with the potential to shorten chemotherapeutic treatment. Ongoing and new drug discovery research initiatives, along with improvements to known classes of compounds, will ensure that the pipeline of new anti-TB compounds continues.

## 10. Concluding Remarks and Perspectives

Pathogenic organisms, such as *M. tuberculosis*, that significantly contribute to worldwide human infectious disease are also the most common antibiotic-resistant bacteria [140]. Our arsenal of antimicrobials is currently under attack by the microorganisms themselves as clinically-significant, antibiotic-resistant bacteria evolve at alarming rates [141]. The fight against antibiotic resistance is formidable, but must be endeavored in the face of treatment failures, prolonged illnesses, increased deaths, and escalated risks of infections. With increases in worldwide cases of MDR- and XDR-TB occurring on a yearly basis, the grim progression from antibiotic effectiveness to antibiotic resistance drives this global crisis. 

The comfort zone of antimicrobial discovery represents a path of proven bacterial targets, such as cell wall biosynthesis, protein synthesis, and nucleic acid metabolism and replication. The development of completely new classes of drugs necessitates new avenues of basic research which incorporate identifying new antimicrobial targets and discovering unique mechanisms of action using novel approaches. However, ensuring TB drug compliance and susceptibility testing is critical, since the introduction of new antibiotics could, ironically and unfortunately, generate additional antibiotic resistance and further intensify the existing problem. Nevertheless, aggressive strategies and innovative approaches are desperately needed to fight XDR-TB or we are likely to lose our grip on TB control and witness the emergence of completely drug-resistant TB.
